# Hypoxia Stimulates Synthesis of Neutrophil Gelatinase-Associated Lipocalin in Aortic Valve Disease

**DOI:** 10.3389/fcvm.2019.00156

**Published:** 2019-10-29

**Authors:** Ganesh Swaminathan, Varun K. Krishnamurthy, Swetha Sridhar, Denise C. Robson, Yao Ning, K. Jane Grande-Allen

**Affiliations:** Department of Bioengineering, Rice University, Houston, TX, United States

**Keywords:** aortic valve disease, hypoxia, valve interstitial cells, neutrophil gelatinase-associated lipocalin, elastin

## Abstract

**Objective:** Aortic valve disease is commonly found in the elderly population. It is characterized by dysregulated extracellular matrix remodeling followed by extensive microcalcification of the aortic valve and activation of valve interstitial cells. The mechanism behind these events are largely unknown. Studies have reported expression of hypoxia inducible factor-1 alpha (HIF1α) in calcific nodules in aortic valve disease, therefore we investigated the effect of hypoxia on extracellular matrix remodeling in aged aortic valves.

**Approach and Results:** Western blotting revealed elevated expression of HIF1α and the complex of matrix metalloprotease 9 (MMP9) and neutrophil gelatinase-associated lipocalin (NGAL) in aged porcine aortic valves cultured under hypoxic conditions. Consistently, immunofluorescence staining showed co-expression of MMP9 and NGAL in the fibrosa layer of these porcine hypoxic aortic valves. Gelatinase zymography demonstrated that the activity of MMP9-NGAL complex was significantly increased in aortic valves in 13% O_2_ compared to 20% O_2_. Importantly, the presence of ectopic elastic fibers in the fibrosa of hypoxic aortic valves, also detected in human diseased aortic valves, suggests altered elastin homeostasis due to hypoxia.

**Conclusion:** This study demonstrates that hypoxia stimulates pathological extracellular matrix remodeling via expression of NGAL and MMP9 by valve interstitial cells.

## Introduction

Aortic valve disease (AVD) is one of the most common heart valve diseases, affecting more than 2% of the aged population in the United States (1, 2). Aortic valve replacement either surgically or through transcatheter aortic valve implantation remains the current treatment modality (3). The aortic valve (AV) has a unique three-layered leaflet structure: the fibrosa (outflow, aortic side) made of collagen, ventricularis (inflow, ventricle side) made of elastic fibers, and spongiosa (intermediate layer) made of proteoglycans and glycosaminoglycans. These heterogeneous extracellular matrix (ECM) proteins impart adequate durability, stress relaxation and flexibility to the AV and are regulated by a specialized group of cells called valve interstitial cells (VICs), which are present across all layers of the AV. In adults, VICs remain quiescent; however the cells undergo phenotypic activation in diseased AVs, resulting in altered ECM remodeling through heightened synthesis and activation of matrix metalloproteases (MMPs).

Although aging is a critical risk factor, AVD is an active multistep process of fibrocalcification of the AV resulting in severe stenosis and dysfunction (1). Initiation of AVD is characterized by endothelial injury triggering inflammation and infiltration of immune cells such as macrophages. However, progression of AVD via pathological ECM remodeling occurs as a separate cell-driven process regulated by VICs (1, 4). Notably, activated VICs undergo phenotypic transition into myofibroblasts and osteoblasts by upregulating transforming growth factor-beta (TGFβ) and nuclear factor kappa B (NFκB) pathways that facilitate progression of AVD.

Since AVs are largely avascular and oxygen (O_2_) transfer within valves occurs via passive diffusion, the thickening of valves with aging can cause regions within AVs to turn progressively hypoxic. We recently demonstrated the extent of hypoxia within both aortic and mitral valves under static conditions (5). Additionally, hypoxia inducible factor-1 alpha (HIF1α), a marker of hypoxia, has been shown to be expressed in the calcific nodules in diseased AVs (6, 7). Nevertheless, the role of hypoxia in the onset of AVD remains unknown. While it has been demonstrated that porcine mitral VICs upregulate MMP 2 and 9 in response to hypoxia (8), dysregulation of matrix remodeling due to hypoxia in aortic valves is not clearly understood.

HIF1α is known to upregulate several proteins capable of ECM remodeling. For instance, HIF1α can regulate expression of NFκB (9) and MMPs 2 and 9 (8), as well as neutrophil gelatinase-associated lipocalin (NGAL) (10). In the recent past, NGAL has gained interest for its ability to accelerate ECM breakdown by interacting with MMP9 and has thus been investigated as a potential biomarker of chronic kidney disease, cancer, and cardiovascular diseases. Therefore, the goal of this study was to determine if hypoxia drives ECM remodeling in adult AVs. Specifically, we tested the hypothesis that hypoxia stimulates expression of NFκB as well as NGAL, which result in ECM remodeling in AVD. Additionally, we also tested hypoxia-mediated activation of the TGFβ pathway via activation of both Smad2/3 (canonical) and mitogen activated protein kinase cascades (non-canonical) to induce ECM remodeling in AVs. We show for the first time that hypoxia upregulated MMP9-NGAL complex and NFκB in cultured porcine AVs as well as pilot HuAVIC cultures. These results provide valuable insights on the role of hypoxia as well as NGAL in valvular ECM remodeling.

## Materials and Methods

### Hypoxic Culture of Porcine AV Leaflets and HuAVICs

Whole AV leaflets from aged porcine hearts (>2 years, *N* = 9) were obtained from a local commercial abattoir (Animal Technologies, Tyler, TX). The AV leaflets were immediately processed for *ex vivo* culture and histology (6/9), while some were preserved in−80°C for protein isolation as fresh tissue controls (3/9). Culture of whole AV leaflets was performed as described previously (5). The leaflets were secured in 6-well plates coated with a 2 mm layer of polydimethylsiloxane (PDMS; Dow Corning, Midland, MI) using a stainless-steel insect pin inserted through the center of each leaflet and the pin tip was secured in PDMS to prevent folding of leaflets. The whole leaflets were cultured for 1 week with culture media (11) containing DMEM (5 mmol/L glucose), Ham's F12 (Hyclone, Logan, UT), 10% v/v bovine growth serum (BGS; Thermo Fisher Scientific, Waltham, MA), and 1% v/v antibiotic (Thermo Fisher Scientific) in either normoxic (20% O_2_) or moderately hypoxic (13% O_2_) conditions since severe hypoxia (5% O_2_) was found to cause significant cell death in both VICs and endothelial cells in AV leaflets cultured *ex vivo* (5). Analysis of protein expression was compared between AVs cultured in 20 vs. 13% O_2_, while the baseline expression was derived from fresh AV controls.

Human hearts with normal AVs were obtained from donors through the National Disease Research Interchange program. All donors (*N* = 4) were over the age 50; they demonstrated normal cardiac structure and function and died of non-cardiac causes. Because these hearts had been considered as prospects for organ donation, they had a warm ischemia time of <6 h prior to their being shipped on ice overnight to Rice University (11). Overtly diseased AVs from patients over the age 50 (*N* = 4) were procured during valve replacement surgeries at the Houston Methodist Hospital (Houston, TX) (6, 12). The valve leaflets from normal, aged and diseased, aged patients were processed for histology. All tissue handling protocols have been approved by the Rice University and the Houston Methodist Hospital Institutional Review Board.

A small pilot analysis was conducted to assess the effects of hypoxia on human aortic valve cells. HuAVICs were isolated from AVs (*N* = 1, healthy, aged >50 years, male patient) using collagenase digests, according to previous published methods (5, 11, 13). Briefly, the whole valve leaflets were cut in half and incubated in 2 mg/ml of collagenase II (Worthington Scientific, Lakewood, NJ) in DMEM containing 2.5% antibiotic/antimycotic (ABAM; Mediatech, Herndon, VA) for 30 min in a shaking incubator at 160 rpm and 37°C and subsequently the loosened endothelial cells were removed by gently brushing the leaflet surface with a sterile cotton swab (14). The tissue was then finely minced and incubated in an enzymatic digestion solution containing collagenase III, hyaluronidase, and neutral protease for 4 h. Subsequently, the mixture was filtered and the cells were pelleted by centrifugation (1,500 × g, 5 min) and resuspended in the media as detailed above. Once confluent, the HuAVICs were passaged, seeded at 100,000 cells per well of a 6-well plate, and then subjected to culture for 1 week in different hypoxic conditions: 20, 13, and 5% O_2_. Media was changed every 2 days.

Hypoxic cultures for both AV leaflets and VICs were conducted in an incubator sub-chamber (BioSpherix, Parish, NY) connected to a gas controller. Briefly, a gas cylinder with custom gas mixes (13 or 5% O_2_, each with 5% carbon dioxide and remaining filled with nitrogen) was connected to the inlet of a pre-programmable O_2_ controller (BioSpherix) and the outlet from oxygen controller was connected to a sub-chamber, which was housed within the traditional cell culture incubator to control temperature (37°C) and humidity. The O_2_ levels (13 or 5%) were maintained throughout the duration of each culture period. Cultures grown under 20% O_2_ were used as controls.

### Immunohistochemistry and Immunofluorescence Staining

To evaluate regional ECM composition and organization, histochemistry was performed on 5 μm tissue sections using Movat's modified pentachrome stain, which colors elastic fibers black, collagen fibers yellow, proteoglycans, and glycosaminoglycans blue, muscle red, and cell nuclei purple. Antibodies directed against remodeling enzymes and TGFβ pathway markers (see Supplementary Table) were used to determine protein expression and localization using immunofluorescence (IF), or streptavidin/biotin colorimetry and diaminobenzidine (DAB) detection (5, 11, 15). Antigen retrieval was performed using heat-mediated citrate buffer. Imaging was performed with confocal and bright-field microscopy. ImageJ (NIH, Bethesda, MD) was used to quantitatively analyze IHC and Movat pentachrome stains, as recently described (5, 16).

### Protein Isolation and Quantification

After 1 week in culture, porcine AV leaflets were harvested for protein isolation as described previously (17, 18). Briefly, the leaflets were flash frozen with liquid nitrogen and stored at −80°C overnight before being lyophilized for 24 h. The lyophilized tissues were harvested in T-PER lysis buffer (Thermo Fisher Scientific) containing 1% Halt™ protease inhibitor cocktail (Thermo Fisher Scientific) and homogenized using a Tissue Lyser II (Qiagen, Germantown, MD). The homogenized tissue lysates were incubated at 4°C for 1 h, then centrifuged at 10,000 × g for 10 min, and the supernatant was collected and stored at −80°C. Likewise, after 1 week in culture, cell lysates from human VICs were collected as described previously (19, 20). The protein content was determined using a bicinchoninic acid protein assay kit (Thermo Fisher Scientific) (20).

### Western Blotting

Hypoxia-induced differential expression of proteins in the AV was semi-quantitatively assessed using western blotting. Briefly, a maximum of 10–20 μl of the tissue or cell lysate was loaded into each lane of a 4–12% bis-tris SDS-PAGE gels (Invitrogen, Carlsbad, CA) under reducing or non-reducing conditions, as described previously (21, 22). The gels were transferred onto nitrocellulose membrane (Bio-Rad, Hercules, CA) using a Trans-Blot semi-dry transfer cell (Bio-Rad), blocked with Li-Cor blocking buffer (LI-COR, Lincoln, NE) for 1 h, and incubated with primary antibodies overnight at 4°C. Primary antibodies against MMPs 2 and 9 (LifeSpan Biosciences, Seattle, WA), NGAL (for porcine AV), HIF1α, TGFβ1 (Abcam, Cambridge, MA), NGAL (for HuAVICs), pSMAD2/3, pERK1/2, and NFκB p65 (Cell Signaling Technologies, Danvers, MA) were used. Either beta-actin (β-actin) or GAPDH (Abcam) were used as loading controls. Secondary antibody labeling was carried out using IRDye® 680LT and IRDye® 800CW antibodies (LI-COR) for 1 h at room temperature. Bands were detected using an Azure cSeries (Azure Biosystems, Dublin, CA) or Odyssey scanning system (LI-COR). Protein band intensities were quantified using Image Studio Lite software (LI-COR) and represented as fold difference compared to respective loading control.

### Zymography

The effect of hypoxia on the enzyme activity of MMPs 2 and 9 was analyzed using gel zymography (18, 20). A total of 10 and 4 μg of tissue and cell lysates, respectively, were loaded in each lane of 10% gelatin zymogram gels (Invitrogen) and run for 2 h at 125 V. Subsequently, the gels were washed with 2.5% v/v TritonX-100 for 30 min, incubated in substrate development buffer (G Biosciences, St. Louis, MO) for 48 h, stained with Coomassie Brilliant Blue (Bio-Rad), and de-stained with water until clear bands were visible. The bands were then visualized using an Azure cSeries scanning system (Azure Biosystems) and quantified as described above.

### Statistical Analysis

The statistical analyses were performed using one-way ANOVA with Tukey's *post-hoc* tests to assess the differences between porcine AVs cultured in 20 vs. 13% O_2_ compared to fresh tissues and for comparisons between HuAVICs cultured in 20, 13, and 5% O_2_. A total of *N* = 3 animals or replicate cultures per condition were analyzed. All values are represented as mean ± standard deviation. Differences were deemed significant for *p* < 0.05. GraphPad Prism software was used for all statistical analyses.

## Results

### Hypoxia Stimulated MMP9-NGAL Complex Expression in Porcine AVs

**Figure 1:**
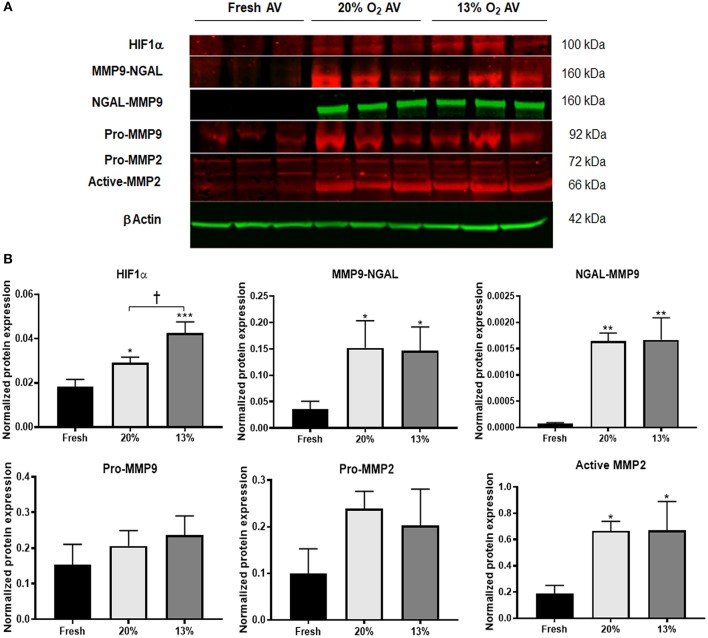
Hypoxia promotes expression of matrix remodeling enzymes in aged (>2 years) porcine aortic valves (AVs) in culture. **(A)** Western blot analysis of tissue lysates from fresh, normoxic (20% O_2_) and hypoxic (13% O_2_) porcine AVs cultured for 1 week. **(B)** Quantification of western blot band intensities using densitometry and normalized to βActin. Data shown are mean ± SD from *N* = 3 animals. ^*^P < 0.05, ^**^P < 0.01, ^***^P < 0.001 vs. fresh AV controls, and ^†^*P* < 0.05 between 20 and 13% O_2_ cultured AVs. Significance determined by one-way ANOVA using Tukey *post-hoc* test. MMP9-NGAL and NGAL-MMP9 represent bands for the complex detected when probed separately for MMP9 and NGAL, respectively.

Since hypoxia was shown to upregulate MMPs 2 and 9 in young AVs, it is of interest to determine the effects of hypoxia on aged AVs, to understand their influence in the initiation and progression of AVD. We first determined whether our hypoxic conditions (13% O_2_) stimulated HIF1α expression. We found that HIF1α had significantly greater expression in cultured porcine AVs relative to fresh AV controls and was the highest in 13% O_2_ AVs compared to 20% O_2_ (Figure 1). Previously, our simulation studies on oxygen diffusion within AVs showed hypoxic regions even under normoxia; indeed, expression of HIF1α in 20% O_2_ likely suggests moderate hypoxia and hence that AVs in 13% O_2_ may experience moderate to severe hypoxia (5). Similarly, expression of active MMP2 was increased in cultured AVs. Different to young AVs, we detected expression of MMP9-NGAL complex (160 kDa), which was significantly higher in aged AVs cultured in both 20 and 13% O_2_ compared to fresh AVs. Furthermore, expression of this complex was also confirmed in cultured AVs by probing separately for NGAL (Figure 1), thus suggesting that hypoxia stimulates expression of MMP9-NGAL in aged AVs.

**Figure 2:**
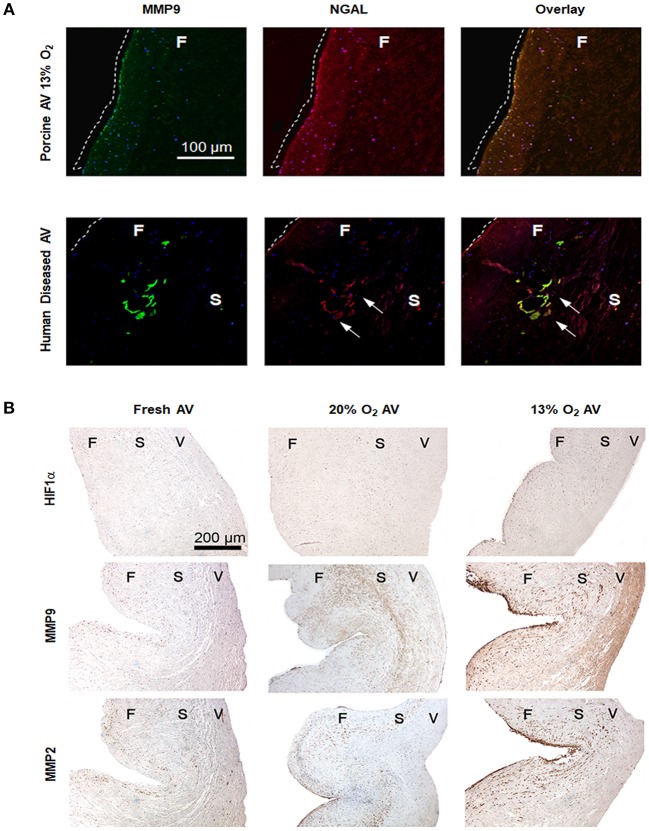
Hypoxia induces expression of matrix metalloproteases (MMPs) 2 and 9 in aged (>2 years) porcine aortic valve (AV) tissue. **(A)** Immunofluorescence staining showing expression of MMP9 and neutrophil gelatinase-associated lipocalin (NGAL) in fibrosa of both porcine AVs cultured in 13% O_2_ and human diseased AV (sclerotic/calcific). Scale bar: 100 μm. Dotted white lines indicate the tissue boundary and arrows indicate expression of NGAL in sclerotic/calcific regions in human AV. **(B)** Immunohistochemistry analysis showing expression of HIF1α, MMPs 2 and 9 in fresh, normoxic (20% O_2_) and hypoxic (13% O_2_) porcine AVs cultured for 1 week. Scale bar: 200 μm. F, fibrosa; S, spongiosa; V, ventricularis.

Although hypoxia is known to stimulate expression of NGAL in cancer and kidney diseases, its expression in AVs or AVD has not been previously reported. To validate if hypoxia stimulated NGAL in AVs and investigate whether NGAL is associated with AVD, we analyzed expression of NGAL in hypoxic porcine and diseased human AVs, respectively. Both NGAL and MMP9 was expressed in the fibrosa of human diseased AVs as well as in hypoxic porcine AVs (Figure 2A), thus suggesting the possible interplay between hypoxia and NGAL in AVD. Additionally, we found increased expression of HIF1α, MMPs2, and 9 in the fibrosa of AVs cultured in 13% O_2_ compared to 20% O_2_ (Figure 2B).

### Hypoxia Stabilized MMP9 Activity via NGAL in Porcine AVs

**Figure 3:**
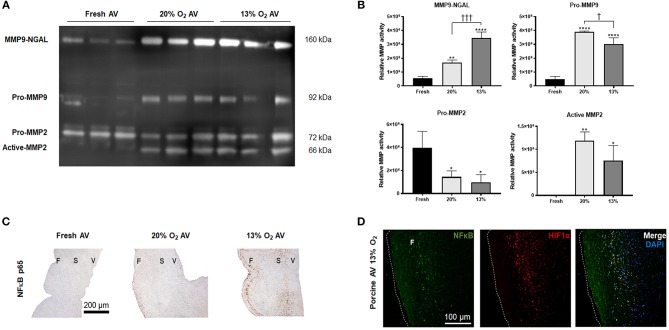
Expression of matrix metalloprotease (MMP) 9 and neutrophil gelatinase-associated lipocalin (NGAL) complex and nuclear factor kappa B (NFκB) in aged (> 2 years) porcine aortic valves (AVs). **(A)** Zymography showing activity of MMP9-NGAL complex, Pro-MMP9, and MMP2 in fresh, normoxic (20% O_2_) and hypoxic (13% O_2_) porcine AVs cultured for 1 week. **(B)** Quantification of band intensities using densitometry. Data shown are mean ± SD from *N* = 3 animals. ^*^ and ^†^ represent significant differences vs. fresh AV controls and between 20 and 13% O_2_ cultured AVs, respectively. ^*^, ^†^*P* < 0.05, ^**^P < 0.01, ^†††^*P* < 0.001, ^****^P < 0.0001 determined by one-way ANOVA using Tukey *post-hoc* test. **(C)** Immunohistochemistry analysis showing expression of NFκB p65 in fresh and cultured porcine AVs in 20, and 13% O_2_. Scale bar: 200 μm. F, fibrosa; S, spongiosa; V, ventricularis. **(D)** Immunofluorescence staining showing co-expression of NFκB and HIF1α in porcine AVs cultured in 13% O_2_. Scale bar: 100 μm.

It is known that NGAL stabilizes MMP9 to promote sustained proteolytic activity; therefore, we investigated the activity of MMP9-NGAL complex in hypoxic AVs. Our results showed significant activity of MMP9-NGAL complex in 13% O_2_ compared to 20% O_2_ (Figure 3). While pro-MMP9 was significantly increased in both AVs in 20 and 13% O_2_, it was the highest in 20% O_2_, thus suggesting that hypoxia may influence both expression as well as activity of MMP9. However, no differences in the activity of MMP2 was observed between the cultured AVs (Figure 3B).

### Expression of NFκB in Hypoxic Porcine AVs

Since NGAL is predominantly upregulated in inflammatory conditions by NFκB (23) and since hypoxia can induce NFκB expression (24), we assessed the expression of NFκB in hypoxic AVs. NFκB p65 was strongly expressed in the fibrosa of 13% O_2_ AVs compared to both 20% O_2_ (Figure 3C). Additionally, our IF results showed co-expression of HIF1α and NFκB p65 within the fibrosa of 13% O_2_ AVs (Figure 3D), suggesting that NFκB activation was likely mediated by HIF1α.

### Dysregulated TGFβ1 Signaling in Cultured Porcine AVs

**Figure 4:**
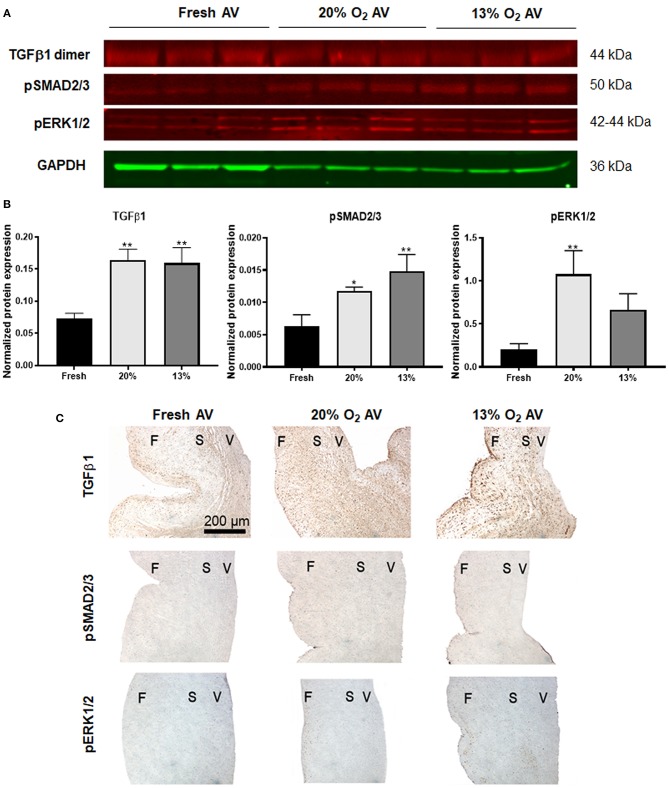
Dysregulation of TGFβ pathway in hypoxic aged (>2 years) porcine aortic valve (AV) tissue. **(A)** Western blot analysis of tissue lysates from fresh, normoxic (20% O_2_) and hypoxic (13% O_2_) porcine AVs cultured for 1 week. **(B)** Quantification of western blot band intensities using densitometry and normalized to GAPDH. Data shown are mean ± SD from *N* = 3 animals. ^*^P < 0.05, ^**^P < 0.01 vs. fresh AV controls, significance determined by one-way ANOVA using Tukey *post-hoc* test. **(C)** Immunohistochemistry analysis showing expression of TGFβ1, pSMAD2/3, and pERK1/2 in fresh and cultured porcine AVs in 20, and 13% O_2_. Scale bar: 200 μm. F, fibrosa; S, spongiosa; V, ventricularis.

Next, we investigated the TGFβ1 pathway, since studies have demonstrated increased expression of active TGFβ1 and its pathway activation factors such as pSMAD2/3 (canonical) and activation of mitogen-activated protein kinase pathway cascade such as the pERK1/2 (non-canonical) in diseased AVs (25, 26). Similarly, we found increased expression of active TGFβ1 (detected as a dimer) as well as pSMAD2/3 in both the cultured AVs, whereas expression of pERK1/2 was significant only in 20% O_2_ (Figures 4A,B), thus suggesting potential dysregulation of TGFβ1 pathway in aged AVs due to hypoxia. These observations were also validated using IHC staining (Figure 4C).

### Altered Elastic Matrix Homeostasis in Hypoxic AVs

**Figure 5:**
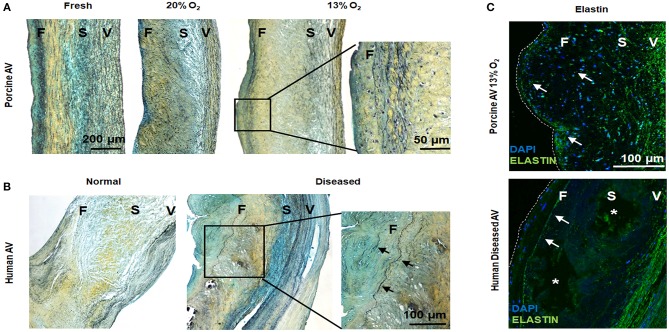
Hypoxic aged (>2 years) porcine aortic valve (AV) and human diseased AV demonstrate ectopic elastic fiber expression in the fibrosa. **(A,B)** Movat histochemical staining in porcine AVs (fresh and cultured in 20 and 13% O_2_ for 1 week) and human normal and diseased (sclerotic/calcified) AVs. Scale bar = 200 μm, 50 μm (top right), and 100 μm (lower right). **(C)** Immunofluorescence staining for elastin (green) on porcine AV cultured in 13% O_2_ for 1 week and human diseased (sclerotic/calcified) AV. F, fibrosa; S, spongiosa; V, ventricularis. Arrows denote elastin expression. ^*^indicate calcific nodule. Scale bar = 100 μm.

While hypoxia is known to regulate collagen as well as glycosaminoglycans in AVs (8, 27), its effects on elastin is not known. We observed fragmented elastic fibers in the ventricularis of both 20 and 13% O_2_ AVs compared to fresh AVs. Importantly, we also visualized ectopic, nascent elastic fibers in the fibrosa of AVs in 13% O_2_ (Figures 5A,C), which is unusual considering that elastin is predominantly observed in the ventricularis. Thus, this suggests the potential role of hypoxia in initiating ECM remodeling in the fibrosa.

To further validate our findings, we investigated diseased human AVs. We found evidences of a thin layer of elastic fiber in the fibrosa in the diseased AVs, whereas fragmented elastic fibers were noted in the ventricularis of both normal and diseased human AVs (Figures 5B,C). Together, these results suggest a link between hypoxia and elastic fiber remodeling in the fibrosa, which may play a role in AVD.

### Pilot Study of Expression of NGAL in Hypoxic Human Aortic VICs

To understand if hypoxia promoted NGAL expression in VICs, we performed a pilot experiment to assess expression of MMP9 and NGAL by HuAVICs isolated from a healthy, aged patient, under 20, 13%, and severely hypoxic 5% O_2_ conditions. While AV leaflets were cultured in moderately hypoxic 13% O_2_, the VICs within these AVs may experience rather severe hypoxic stress, therefore 5% O_2_ conditions were used. Although the results reported here (and shown in the Supplementary Figure 1) are limited in scope as the VICs were from a single human valve, the pilot study was informative. When probed under strong reducing conditions, different to that performed for the porcine AV leaflet samples, we detected no complex of MMP9-NGAL, however the levels of pro-MMP9 and unbound-NGAL was significantly higher in VICs cultured in 5% compared to 20% O_2_ (Supplementary Figure 1A). The MMP-NGAL complex was only detected in the HuAVICs in 5% O_2_ under non-reducing and non-denaturing conditions (Supplementary Figure 1B). Due to the very limited sample size of this pilot analysis, further study will be needed to assess whether severe hypoxia induces expression and stabilization of MMP9-NGAL complex in HuAVICs.

## Discussion

Here, we demonstrated the effects of hypoxia in aged AVs. Given that almost all studies that investigated role of hypoxia in valve disease employed valves from young animals (5, 8, 27), our major goal was to study aged AVs from pigs and humans to gain better insights on the influence of hypoxia on valve disease. We sought to study porcine AVs because pigs have been shown to be a good model to study heart valve diseases. Considering the anatomical and structural similarity to aged humans (60–70 years), porcine AVs (>2 years) were used. The AVs were cultured for 1 week only as we noticed tissue necrosis beyond that time frame.

Hypoxic stress was confirmed by upregulation of HIF1α in AVs cultured in both 13 and 20% O_2_, in agreement with our previous published work (5). Similarly, individual effects of hypoxia on upregulating MMP2, MMP9 expression and activity was found to be consistent with other studies. For the first time, we showed expression of MMP9-NGAL complex in the cultured AV leaflets. The pilot study of VICs was consistent with that result but required a more severe state of experimental hypoxic stress, which we speculate is due to the cells deep within the leaflets being naturally subjected to a hypoxic environment. Several studies in cancer (28–30), kidney (10, 31, 32), and cardiovascular diseases (33–35) have demonstrated association of NGAL with MMP9 to facilitate ECM remodeling. Furthermore, NGAL is secreted in hypoxic conditions primarily by neutrophils or by other cell types including endothelial cells (36), macrophages (36), cardiomyocytes (37), and epithelial cells (38), but no reports on NGAL expression by VICs exists.

Although interaction between NGAL and MMP9 is still not clearly understood, we believe that the complex demonstrated in this work contains the homotrimer NGAL (70 kDa) and pro-MMP9 (28). However, in most studies the complex was cleaved under strong reducing and denaturing conditions (39); our observation of the intact complex under similar conditions suggests that the interaction is likely hydrophobic in aged porcine AVs cultured under hypoxia, as demonstrated by Kiczak et al. (40). Hence, this strong association of NGAL with MMP9 explains the heightened activity of the MMP9-NGAL complex in 13% O_2_.

The general consensus that AVD originates in the fibrosa, since it is the most affected layer during AVD (41, 42), was supported by our IHC results. Analysis of HIF1α, MMP2, and MMP9 as well as the co-staining of MMP9 and NGAL showed strong expression in the fibrosa of AVs in 13% O_2_. Furthermore, given that NFκB as well as HIF1α can stimulate synthesis of NGAL (10), we believe that the increased expression of NGAL in AVs was likely mediated by NFκB/HIF1α pathway, which was also found to be co-expressed in the fibrosa of AVs in 13% O_2_. Another major signaling cascade implicated in AVD is the TGFβ pathway. TGFβ pathways regulate VIC phenotype as well as ECM remodeling via activation of the components in the pathway (43, 44). Our results showed increased expression of active TGFβ1, pSMAD2/3, and pERK1/2 in the cultured AVs, thus suggesting possible dysregulation of the TGFβ pathway under hypoxic stress.

Since hypoxia upregulated the activity of MMP9-NGAL complex, we investigated its effects on valvular elastin remodeling. Our findings of ectopic elastic fibers in the fibrosa of 13% O_2_ AVs as well as human diseased AVs suggest increased crosslinking of the elastin monomers by lysyl oxidase, an enzyme found to be increased under hypoxic conditions (45, 46). Therefore, together with the increased expression of MMP9 and NGAL in the fibrosa, our results confirmed the association of hypoxia in elastic fiber remodeling in AVD and implicates NGAL as a potential marker for initiation of AVD. Our future studies will expand upon this outcome to delineate the mechanism of hypoxia-mediated VIC stimulation toward AVD using leaflets and quiescent VICs isolated from several human donor hearts.

Overall, the current study demonstrates the influence of hypoxia on the pathological remodeling in aged AVs possibly by upregulating expression and activity of MMP9-NGAL. The findings in this study will provide insights into understanding the role of NGAL on AVD pathogenesis.

## Data Availability Statement

All datasets generated for this study are included in the article/Supplementary Material.

## Ethics Statement

The studies involving human participants were reviewed and approved by Rice University and Houston Methodist Hospital Institutional Review Board. The patients/participants provided their written informed consent to participate in this study.

## Author Contributions

GS and VK conceived and designed the experiments. GS, VK, SS, DR, and YN performed experiments and data analysis. GS wrote the manuscript. KG-A discussed the experimental design, results with the other authors, edited, and approved the manuscript.

### Conflict of Interest

The authors declare that the research was conducted in the absence of any commercial or financial relationships that could be construed as a potential conflict of interest.

## References

[1] Thanassoulis G. Lipoprotein (a) in calcific aortic valve disease: from genomics to novel drug target for aortic stenosis. J Lipid Res. (2016) 57:917–24. 10.1194/jlr.R051870 26685327 PMC4878194

[2] O'Brien KD. Pathogenesis of calcific aortic valve disease: a disease process comes of age (and a good deal more). Arterioscler Thromb Vasc Biol. (2006) 26:1721–8. 10.1161/01.ATV.0000227513.13697.ac 16709942

[3] Brown JM O'Brien SM Wu C Sikora JA Griffith BP Gammie JS. Isolated aortic valve replacement in North America comprising 108,687 patients in 10 years: changes in risks, valve types, and outcomes in the Society of Thoracic Surgeons National Database. J Thorac Cardiovasc Surg. (2009) 137:82–90. 10.1016/j.jtcvs.2008.08.015 19154908

[4] Chen JH Simmons CA. Cell-matrix interactions in the pathobiology of calcific aortic valve disease: critical roles for matricellular, matricrine, and matrix mechanics cues. Circ Res. (2011) 108:1510–24. 10.1161/CIRCRESAHA.110.234237 21659654

[5] Sapp MC Krishnamurthy VK Puperi DS Bhatnagar S Fatora G Mutyala N. Differential cell-matrix responses in hypoxia-stimulated aortic versus mitral valves. J R Soc Interface. (2016) 13:20160449. 10.1098/rsif.2016.0449 28003526 PMC5221519

[6] Stephens EH Saltarrelli JG Jr Balaoing LR Baggett LS Nandi I Anderson KM. Hyaluronan turnover and hypoxic brown adipocytic differentiation are co-localized with ossification in calcified human aortic valves. Pathol Res Pract. (2012) 208:642–50. 10.1016/j.prp.2012.08.001 23017666 PMC3496006

[7] Perrotta I Moraca FM Sciangula A Aquila S Mazzulla S. HIF-1alpha and VEGF: immunohistochemical profile and possible function in human aortic valve stenosis. Ultrastruct Pathol. (2015) 39:198–206. 10.3109/01913123.2014.991884 25569379

[8] Salhiyyah K Sarathchandra P Latif N Yacoub MH Chester AH. Hypoxia-mediated regulation of the secretory properties of mitral valve interstitial cells. Am J Physiol Heart Circ Physiol. (2017) 313:H14–23. 10.1152/ajpheart.00720.2016 28314761

[9] D'Ignazio L Bandarra D Rocha S. NF-κB and HIF crosstalk in immune responses. FEBS J. (2016) 283:413–24. 10.1111/febs.13578 26513405 PMC4864946

[10] Viau A El Karoui K Laouari D Burtin M Nguyen C Mori K. Lipocalin 2 is essential for chronic kidney disease progression in mice and humans. J Clin Invest. (2010) 120:4065–76. 10.1172/JCI42004 20921623 PMC2964970

[11] Krishnamurthy VK Stout AJ Sapp MC Matuska B Lauer ME Grande-Allen KJ. Dysregulation of hyaluronan homeostasis during aortic valve disease. Matrix Biol. (2017) 62:40–57. 10.1016/j.matbio.2016.11.003 27856308 PMC10615645

[12] Stephens EH Saltarrelli JG Baggett LS Nandi I Kuo JJ Davis AR. Differential proteoglycan and hyaluronan distribution in calcified aortic valves. Cardiovasc Pathol. (2011) 20:334–42. 10.1016/j.carpath.2010.10.002 21185747 PMC3075347

[13] Stephens EH Carroll JL Grande-Allen KJ. The use of collagenase III for the isolation of porcine aortic valvular interstitial cells: rationale and optimization. J Heart Valve Dis. (2007) 16:175–83. 17484468

[14] Blevins TL Carroll JL Raza AM Grande-Allen KJ. Phenotypic characterization of isolated valvular interstitial cell subpopulations. J Heart Valve Dis. (2006) 15:815–822. 17152790

[15] Shojaee M Swaminathan G Bashur CA Ramamurthi A. Temporal changes in peritoneal cell phenotype and neoelastic matrix induction with hyaluronan oligomers and TGF-beta1 after implantation of engineered conduits. J Tissue Eng Regen Med. (2018) 12:1420–31. 10.1002/term.2674 29701914

[16] Balaoing LR Post AD Liu H Minn KT Grande-Allen KJ. Age-related changes in aortic valve hemostatic protein regulation. Arterioscler Thromb Vasc Biol. (2014) 34:72–80. 10.1161/ATVBAHA.113.301936 24177329 PMC4685477

[17] Dahal S Broekelman T Mecham RP Ramamurthi A. Maintaining elastogenicity of mesenchymal stem cell-derived smooth muscle cells in two-dimensional culture. Tissue Eng Part A. (2018) 24:979–89. 10.1089/ten.tea.2017.0237 29264957 PMC5984560

[18] Swaminathan G Sivaraman B Moore L Zborowski M Ramamurthi A. Magnetically responsive bone marrow mesenchymal stem cell-derived smooth muscle cells maintain their benefits to augmenting elastic matrix neoassembly. Tissue Eng Part C Methods. (2016) 22:301–11. 10.1089/ten.tec.2015.0349 26830683 PMC4827279

[19] Swaminathan G Stoilov I Broekelmann T Mecham R Ramamurthi A. Phenotype-based selection of bone marrow mesenchymal stem cell-derived smooth muscle cells for elastic matrix regenerative repair in abdominal aortic aneurysms. J Tissue Eng Regen Med. (2018) 12:e60–70. 10.1002/term.2349 27860330

[20] Swaminathan G Gadepalli VS Stoilov I Mecham RP Rao RR Ramamurthi A. Pro-elastogenic effects of bone marrow mesenchymal stem cell-derived smooth muscle cells on cultured aneurysmal smooth muscle cells. J Tissue Eng Regen Med. (2017) 11:679–93. 10.1002/term.1964 25376929

[21] Li Y Wen Y Wang Z Wei Y Wani P Green M. Smooth muscle progenitor cells derived from human pluripotent stem cells induce histologic changes in injured urethral sphincter. Stem Cells Transl Med. (2016) 5:1719–29. 10.5966/sctm.2016-0035 27460854 PMC5189655

[22] Sivaraman B Swaminathan G Moore L Fox J Seshadri D Dahal S. Magnetically-responsive, multifunctional drug delivery nanoparticles for elastic matrix regenerative repair. Acta Biomater. (2017) 52:171–86. 10.1016/j.actbio.2016.11.048 27884774

[23] Iannetti A Pacifico F Acquaviva R Lavorgna A Crescenzi E Vascotto C. The neutrophil gelatinase-associated lipocalin (NGAL), a NF-κB-regulated gene, is a survival factor for thyroid neoplastic cells. Proc Natl Acad Sci USA. (2008) 105:14058–63. 10.1073/pnas.0710846105 18768801 PMC2544578

[24] D'Ignazio L Rocha S. Hypoxia Induced NF-κB. Cells. (2016) 5:10. 10.3390/cells5010010 27005664 PMC4810095

[25] Anger T El-Chafchak J Habib A Stumpf C Weyand M Daniel WG. Statins stimulate RGS-regulated ERK 1/2 activation in human calcified and stenotic aortic valves. Exp Mol Pathol. (2008) 85:101–11. 10.1016/j.yexmp.2008.06.002 18671964

[26] Osman N Grande-Allen KJ Ballinger ML Getachew R Marasco S O'Brien KD. Smad2-dependent glycosaminoglycan elongation in aortic valve interstitial cells enhances binding of LDL to proteoglycans. Cardiovasc Pathol. (2013) 22:146–55. 10.1016/j.carpath.2012.07.002 22999704 PMC10584518

[27] Amofa D Hulin A Nakada Y Sadek HA Yutzey KE. Hypoxia promotes primitive glycosaminoglycan-rich extracellular matrix composition in developing heart valves. Am J Physiol Heart Circ Physiol. (2017) 313:H1143–54. 10.1152/ajpheart.00209.2017 28842437 PMC5814654

[28] Bouchet S Bauvois B. Neutrophil gelatinase-associated lipocalin (NGAL), pro-matrix metalloproteinase-9 (pro-MMP-9) and their complex Pro-MMP-9/NGAL in leukaemias. Cancers. (2014) 6:796–812. 10.3390/cancers6020796 24713998 PMC4074804

[29] Yang J Moses MA. Lipocalin 2: a multifaceted modulator of human cancer. Cell Cycle. (2009) 8:2347–52. 10.4161/cc.8.15.9224 19571677 PMC3381736

[30] Cramer EP Glenthoj A Hager M Juncker-Jensen A Engelholm LH Santoni-Rugiu E. No effect of NGAL/lipocalin-2 on aggressiveness of cancer in the MMTV-PyMT/FVB/N mouse model for breast cancer. PLoS ONE. (2012) 7:e39646. 10.1371/journal.pone.0039646 22737251 PMC3380857

[31] Schmidt-Ott KM Mori K Li JY Kalandadze A Cohen DJ Devarajan P. Dual action of neutrophil gelatinase-associated lipocalin. J Am Soc Nephrol. (2007) 18:407–13. 10.1681/ASN.2006080882 17229907

[32] Ko GJ Grigoryev DN Linfert D Jang HR Watkins T Cheadle C. Transcriptional analysis of kidneys during repair from AKI reveals possible roles for NGAL and KIM-1 as biomarkers of AKI-to-CKD transition. Am J Physiol Renal Physiol. (2010) 298:F1472–83. 10.1152/ajprenal.00619.2009 20181666

[33] Marques FZ Prestes PR Byars SG Ritchie SC Wurtz P Patel SK. Experimental and Human evidence for lipocalin-2 (neutrophil gelatinase-associated lipocalin [NGAL]) in the development of cardiac hypertrophy and heart failure. J Am Heart Assoc. (2017) 6:e005971. 10.1161/JAHA.117.005971 28615213 PMC5669193

[34] Sivalingam Z Larsen SB Grove EL Hvas AM Kristensen SD Magnusson NE. Neutrophil gelatinase-associated lipocalin as a risk marker in cardiovascular disease. Clin Chem Lab Med. (2017) 56:5–18. 10.1515/cclm-2017-0120 28672731

[35] Eilenberg W Stojkovic S Kaider A Kozakowski N Domenig CM Burghuber C. NGAL and MMP-9/NGAL as biomarkers of plaque vulnerability and targets of statins in patients with carotid atherosclerosis. Clin Chem Lab Med. (2017) 56:147–56. 10.1515/cclm-2017-0156 28672747

[36] Eilenberg W Stojkovic S Piechota-Polanczyk A Kaun C Rauscher S Groger M. Neutrophil gelatinase-associated lipocalin (NGAL) is associated with symptomatic carotid atherosclerosis and drives pro-inflammatory state *in vitro*. Eur J Vasc Endovasc Surg. (2016) 51:623–31. 10.1016/j.ejvs.2016.01.009 26947538

[37] Yndestad A Landro L Ueland T Dahl CP Flo TH Vinge LE. Increased systemic and myocardial expression of neutrophil gelatinase-associated lipocalin in clinical and experimental heart failure. Eur Heart J. (2009) 30:1229–36. 10.1093/eurheartj/ehp088 19329498

[38] Kjeldsen L Cowland JB Borregaard N. Human neutrophil gelatinase-associated lipocalin and homologous proteins in rat and mouse. Biochim Biophys Acta. (2000) 1482:272–83. 10.1016/S0167-4838(00)00152-7 11058768

[39] Wei T Zhang H Cetin N Miller E Moak T Suen JY Richter GT Elevated expression of matrix metalloproteinase-9 not matrix metalloproteinase-2 contributes to progression of extracranial arteriovenous malformation. Sci Rep. (2016) 6:24378 10.1038/srep24378 27075045 PMC4830979

[40] Kiczak L Tomaszek A Bania J Paslawska U Zacharski M Noszczyk-Nowak A Expression and complex formation of MMP9, MMP2, NGAL, and TIMP1 in porcine myocardium but not in skeletal muscles in male pigs with tachycardia-induced systolic heart failure. Biomed Res Int. (2013) 2013:283856 10.1155/2013/283856 23710440 PMC3654659

[41] Otto CM Kuusisto J Reichenbach DD Gown AM O'Brien KD. Characterization of the early lesion of 'degenerative' valvular aortic stenosis. Histological and immunohistochemical studies. Circulation. (1994) 90:844–53. 10.1161/01.CIR.90.2.844 7519131

[42] Weinberg EJ Kaazempur Mofrad MR. Transient, three-dimensional, multiscale simulations of the human aortic valve. Cardiovasc Eng. (2007) 7:140–55. 10.1007/s10558-007-9038-4 18026835

[43] Conway SJ Doetschman T Azhar M. The inter-relationship of periostin, TGF beta, and BMP in heart valve development and valvular heart diseases. Sci World J. (2011) 11:1509–24. 10.1100/tsw.2011.132 21805020 PMC5548286

[44] Gu X Masters KS. Role of the MAPK/ERK pathway in valvular interstitial cell calcification. Am J Physiol Heart Circ Physiol. (2009) 296:H1748–57. 10.1152/ajpheart.00099.2009 19363136 PMC5243218

[45] Ji F Wang Y Qiu L Li S Zhu J Liang Z. Hypoxia inducible factor 1alpha-mediated LOX expression correlates with migration and invasion in epithelial ovarian cancer. Int J Oncol. (2013) 42:1578–88. 10.3892/ijo.2013.1878 23545606 PMC3661201

[46] Xie Q Xie J Tian T Ma Q Zhang Q Zhu B. Hypoxia triggers angiogenesis by increasing expression of LOX genes in 3-D culture of ASCs and ECs. Exp Cell Res. (2017) 352:157–63. 10.1016/j.yexcr.2017.02.011 28189640

