# Supplementary material for: Hypoxia Stimulates Synthesis of Neutrophil Gelatinase-Associated Lipocalin in Aortic Valve Disease
Source: Front Cardiovasc Med. 2019 Oct 29;6:156. doi: 10.3389/fcvm.2019.00156 (PMC6828964; doi:10.3389/fcvm.2019.00156)
Supplement: Supplementary file 1 [file Table_1.DOCX]

**SUPPLEMENTARY TABLE**

**Antibodies**

| **Target antigen** | **Vendor or Source** | **Catalog #** | **Working concentration** |
| --- | --- | --- | --- |
| HIF1α | Abcam | ab 179483 | 1: 500, 1:50 for IHC |
| MMP9 | LSBio | LS C31757 | 1: 350, 1:200 for IHC |
| MMP2 | LSBio | LS C314391 | 1: 350, 1:200 for IHC |
| NGAL | Abcam | ab 188551 | 1: 250, 1:25 (human IHC) |
| NGAL (against human) | Cell Signaling | 44058S | 1: 500 |
| NFκB p65 | Cell Signaling | 6956T | 1: 1000, 1:100 for IHC |
| β Actin (Mouse monoclonal) | Abcam | ab 8224 | 1: 1000 |
| β Actin (Rabbit polyclonal) | Abcam | Ab 8227 | 1: 1000 |
| TGFβ1 | Abcam | Ab 92486 | 1: 250, 1:200 for IHC |
| pSMAD2/3 | Cell Signaling | 8828S | 1: 500, 1:200 for IHC |
| pERK1/2 | Cell Signaling | 9101S | 1: 500, 1:200 for IHC |
| Tropoelastin | Robert Mecham |  | 1:100 for IHC |
| IRDye 680LT | LI-COR Biosciences | 925-68021 | 1: 15000 |
| IRDye 800CW | LI-COR Biosciences | 925-32210 | 1: 20000 |

**Cultured Cells**

| **Name** | **Vendor or Source** | **Sex (F, M, or unknown)** |
| --- | --- | --- |
| Human aortic valve interstitial cells | Healthy donor | M |
