# Supplementary figures and images for: Hypoxia Stimulates Synthesis of Neutrophil Gelatinase-Associated Lipocalin in Aortic Valve Disease
Source: Front Cardiovasc Med. 2019 Oct 29;6:156. doi: 10.3389/fcvm.2019.00156 (PMC6828964; doi:10.3389/fcvm.2019.00156)

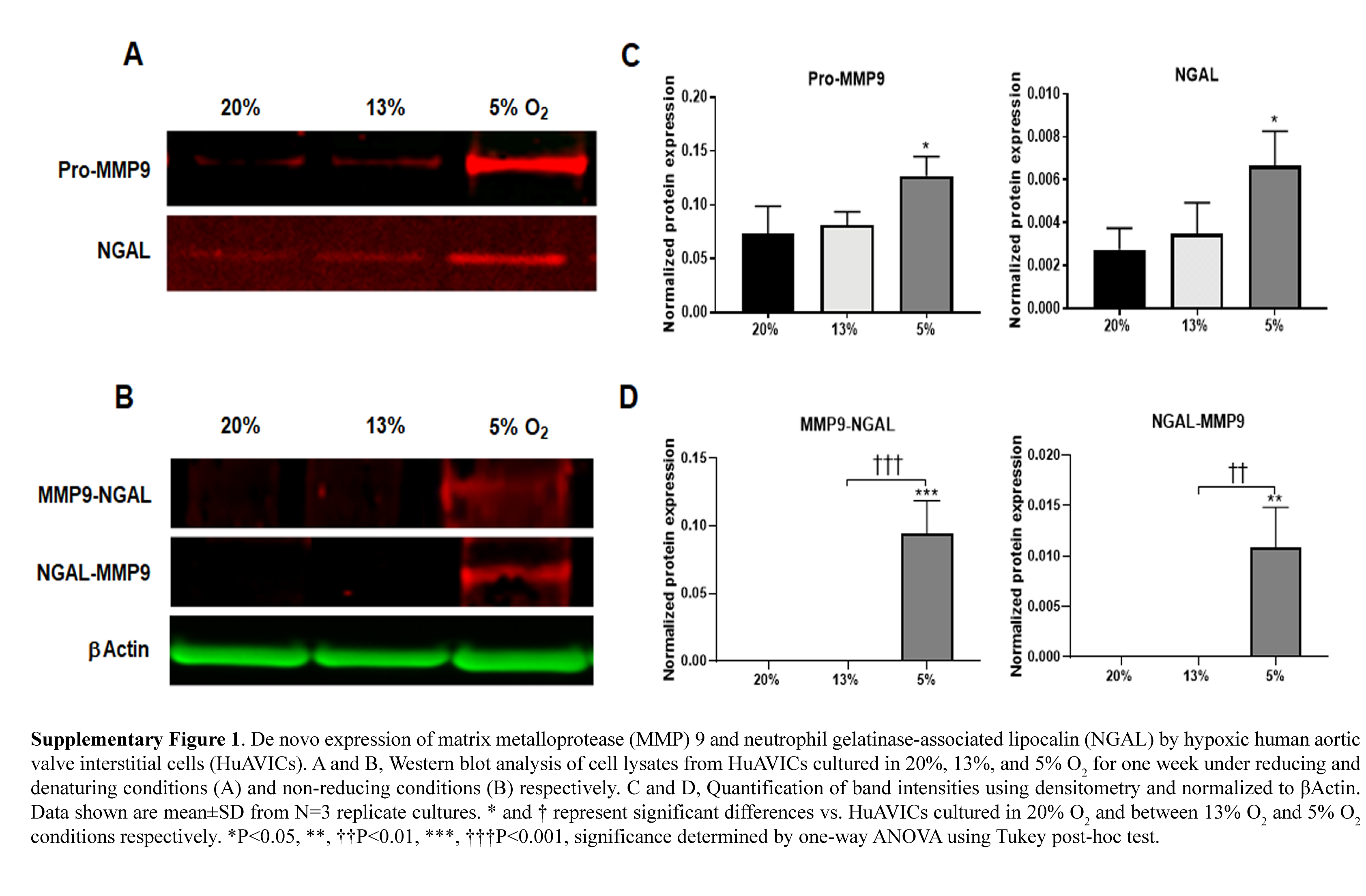

Supplement: Supplementary file 2 [file Image_1.TIF]
